# Supplementary material for: Cost-effectiveness of dengue vaccination in Puerto Rico
Source: PLoS Negl Trop Dis. 2021 Jul 26;15(7):e0009606. doi: 10.1371/journal.pntd.0009606 (PMC8341694; doi:10.1371/journal.pntd.0009606)
Supplement: S1 Text — (DOCX) [file pntd.0009606.s001.docx]

## SUPPORTING INFORMATION

## Sensitivity analysis

### Higher transmission setting

We explored a scenario in which Puerto Rico has a higher transmission intensity of $PE_{9}=60\text{\%}$. In this scenario, we found that around five hospitalizations in naïve children occurred for every 1,000 people vaccinated, compared to six hospitalizations with $PE_{9}=50\text{\%}$ (Figure [S1](#fig-extra-neghosp-60)). Compared to the baseline scenario, we found a slight increase in the number of hospitalizations averted for each additional hospitalization in the naïve vaccinated group. However, in the scenario of higher specificity (0.99) with lower sensitivity (0.64), we found that this proportion of hospitalizations averted almost tripled, resulting in 141 (84 - 813) hospitalizations averted for each additional hospitalization in the naïve vaccinees group.

We found that the intervention was slightly more cost-effective in a higher transmission setting of $PE_{9}=60\text{\%}$ (Figure [S2](#fig-ICER-60)). The ICER of the intervention was around 90,000 USD per QALY averted, which represented a reduction of 18,000 USD from the baseline scenario of $PE_{9}=50\text{\%}$. In terms of symptomatic cases and hospitalizations, the ICER was also lower. We estimated an ICER of 8,000 USD per symptomatic case averted, and 11,000 USD per hospitalization averted.

### Sensitivity of cost-effectiveness to uncertainty in sensitivity and specificity values

The cost-effectiveness ratio of the intervention increased in a scenario of lower specificity (0.76) with higher sensitivity (0.95). The increase was higher in a low-transmission scenario, in part because a reduction in specificity in such a low-transmission level implied a lower number of hospitalizations averted and a higher proportion of hospitalizations caused by misclassification. In contrast, increasing the specificity while reducing sensitivity, slightly reduced the ICER. Changes in the sensitivity and specificity of serological screening did not affect substantially the cost to avert a symptomatic case. With an assumption of lower specificity, more hospitalized cases occurred in the lower transmission scenario, increasing the cost to avert a hospitalization to around 66,000 USD. The cost to avert a hospitalization also increased in a moderate transmission setting to around 24,000 USD. Finally, increasing specificity at a lower sensitivity reduced the cost per hospitalization averted to around 29,000 USD for a low transm

### Lower coverage

Achieving 80% coverage of the intervention (i.e., serological screening and vaccination in the event of a positive result) in 9-year-olds might be unfeasible. We explored a scenario with a lower coverage of 50% to estimate the effects on cost-effectiveness at a lower vaccination coverage. We found that a lower coverage of vaccination increases slightly the incremental cost of gaining a QALY in a moderate transmission setting ($PE_{9}=50\text{\%}$)(Fig. [S4](#fig-ICER-low-cov), left panel). Lower coverage also slightly increased the cost to avert a symptomatic case. In contrast, lower coverage had a minimal impact on the cost-effectiveness to avert a hospitalization case (Fig. [S4](#fig-ICER-low-cov)). Although we assume that the vaccine does not provide permanent protection against infection, the slight difference in the cost-effectiveness at lower coverage could be explained by the temporary cross-protection acquired from vaccination, resulting in indirect protection from vaccination in the short term. The overall magnitude of this indirect protection increases with coverage, as more people acquire temporary protection from infection. Given that some cases would be prevented from this indirect protection, in a moderate transmission setting the high-coverage scenario has slightly lower cost per averted symptomatic case than the low-coverage scenario.

### Uncertainty about costs and disutility values

To reflect uncertainty on the assumptions of treatment costs and disutility, we varied the costs of hospitalized and symptomatic cases by 20%, and used the uncertainty intervals on disutility values from Zeng et al. [26] (Table [S1](#table-psa-costs-qaly)). Reducing the costs of hospitalization 20% below the baseline assumption resulted in an increase of around 125,000 USD per QALY gained in the ICER, while an increment of 20% reduced the ICER to 119,000 USD. Estimates of the ICER showed little sensitivity to 20% variation of the cost of clinical attention of symptomatic cases (121,000 USD - 122,500 USD). Uncertainty on the disutility values of symptomatic cases resulted in a difference of around 62,000 USD in the ICER, while the uncertainty on the disutility of hospitalizations resulted in a 44,000 USD difference in the ICER. Similar magnitudes were found for the sensitivity of the ICER estimates at lower transmission intensity. Given the large uncertainty associated with the cost of serological screening, we assumed a wide range of values with a lower bound of the unit cost of serological screening of 1 USD, and upper bound of 60 USD. The ICER on these upper and lower bounds showed that increasing the cost to 60 USD increased the ICER to 143,000 USD, whereas the ICER would be reduced to 101,000 USD with an assumption of 1 USD.
