# Supplementary figures and images for: Cost-effectiveness of dengue vaccination in Puerto Rico
Source: PLoS Negl Trop Dis. 2021 Jul 26;15(7):e0009606. doi: 10.1371/journal.pntd.0009606 (PMC8341694; doi:10.1371/journal.pntd.0009606)

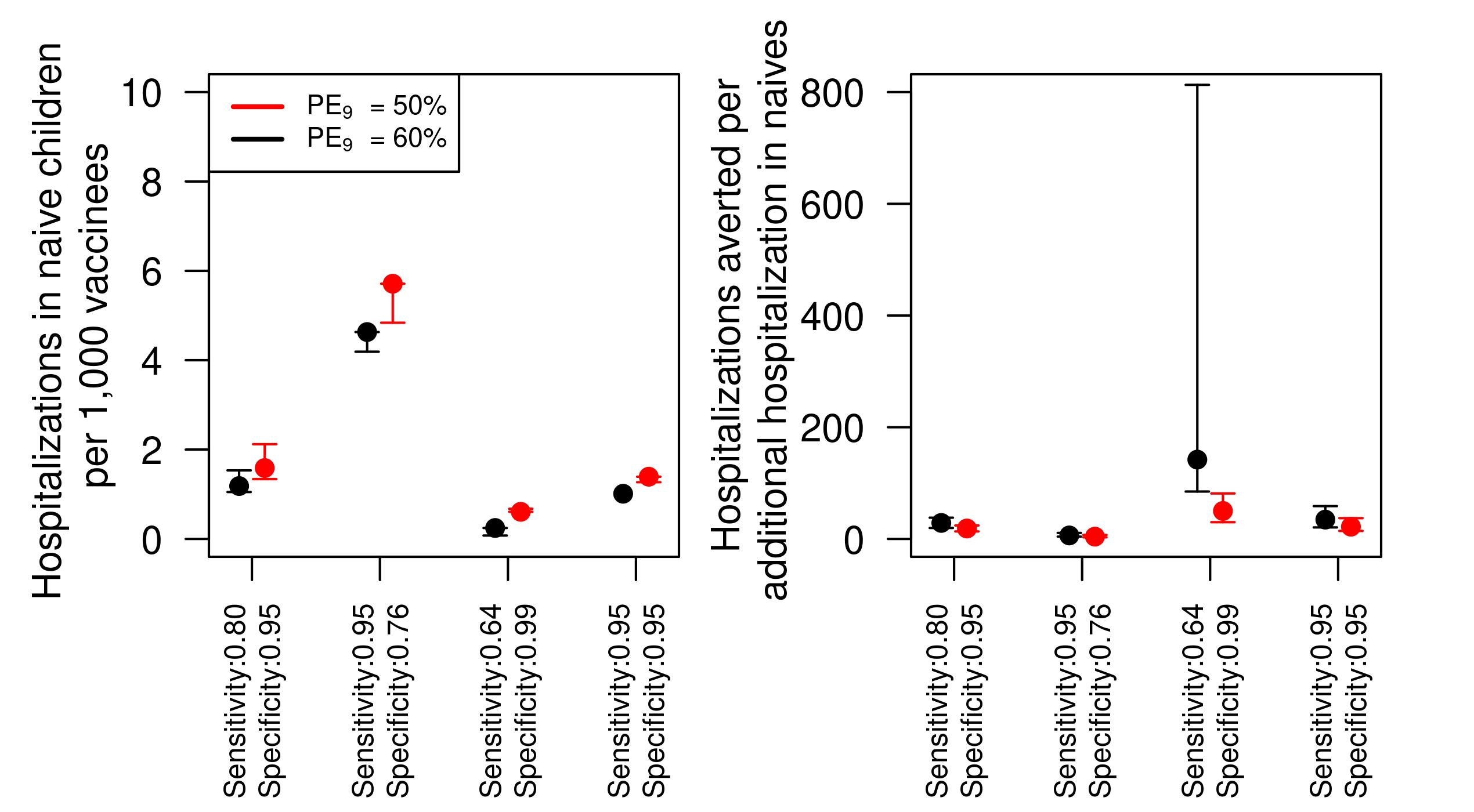

Supplement: S1 Fig — Left panel shows the number of hospitalizations per every 1,000 children vaccinated. The right panel shows the number of hospitalizations averted for every additional hospitalization case in the DENV-naïve group. The simulations were performed for 80% intervention coverage of routine pre-vaccination screening in 9 year-olds over 10 years. (TIF) [file pntd.0009606.s002.tif]

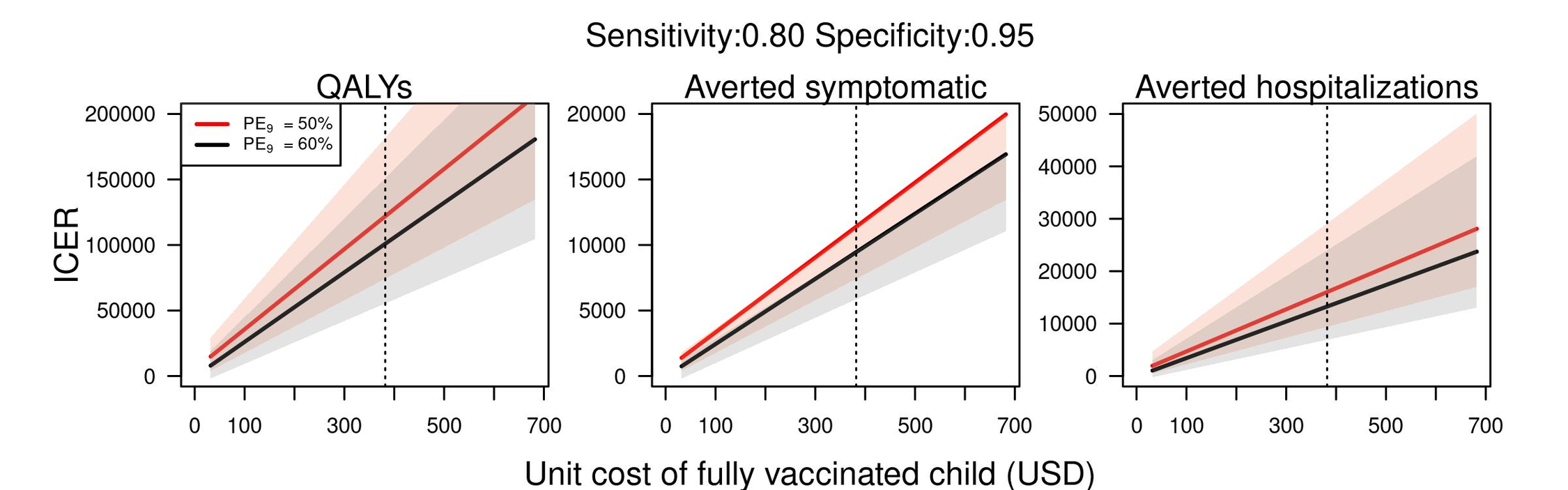

Supplement: S2 Fig — ICER of pre-vaccination screening strategy in Puerto Rico with a higher transmission setting (PE9 = 60%) at different costs of vaccination (total cost for three doses per person), assuming a unit cost of serological screening of 30 USD. Red lines represent a transmission intensity scenario of PE9 = 50%, and black lines represent a transmission scenario of PE9 = 30%. All costs in 2019 USD. (TIF) [file pntd.0009606.s003.tif]

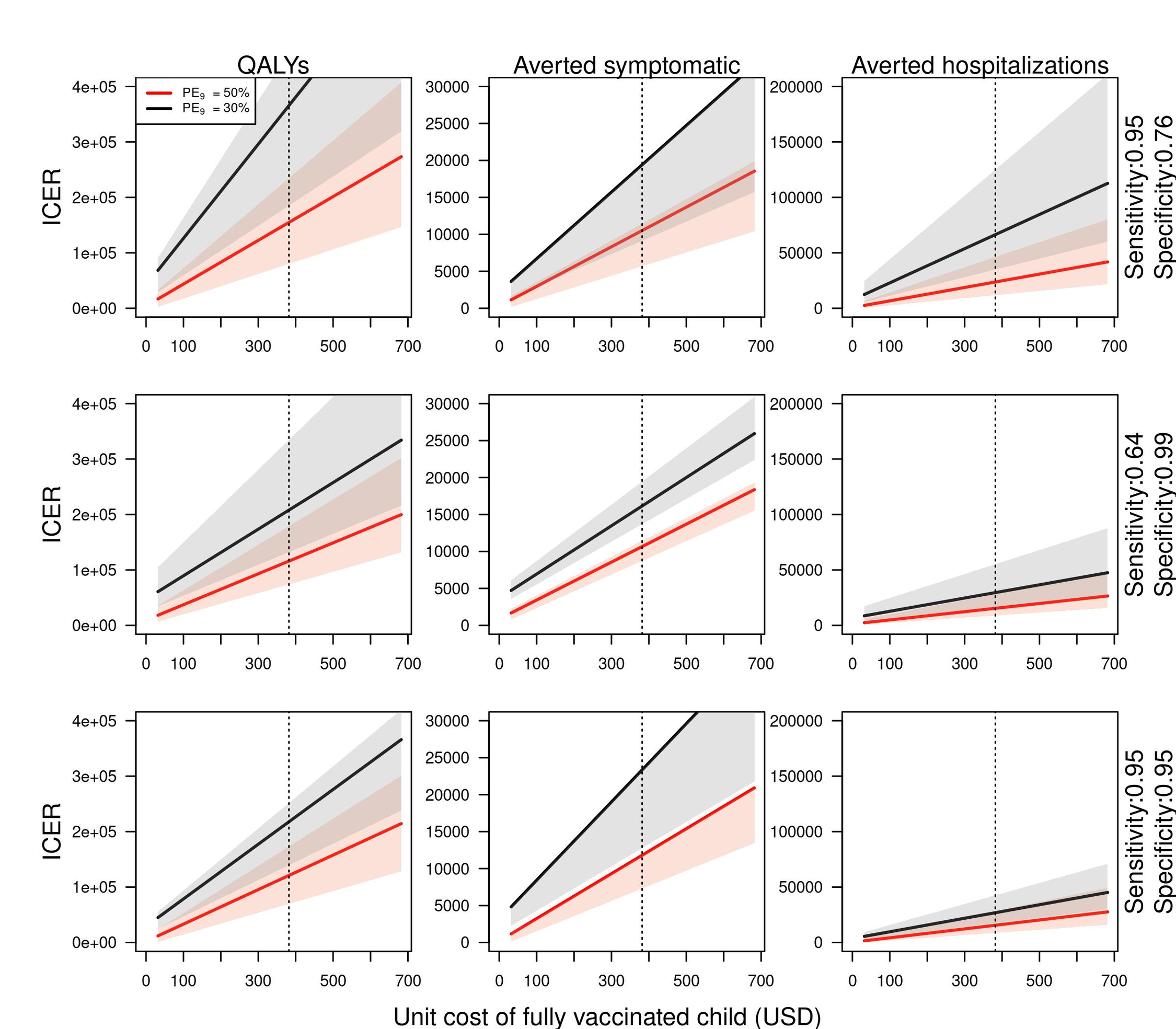

Supplement: S3 Fig — Dotted vertical line represents the baseline cost of vaccination (382 USD). All costs in 2019 USD. (TIF) [file pntd.0009606.s004.tif]

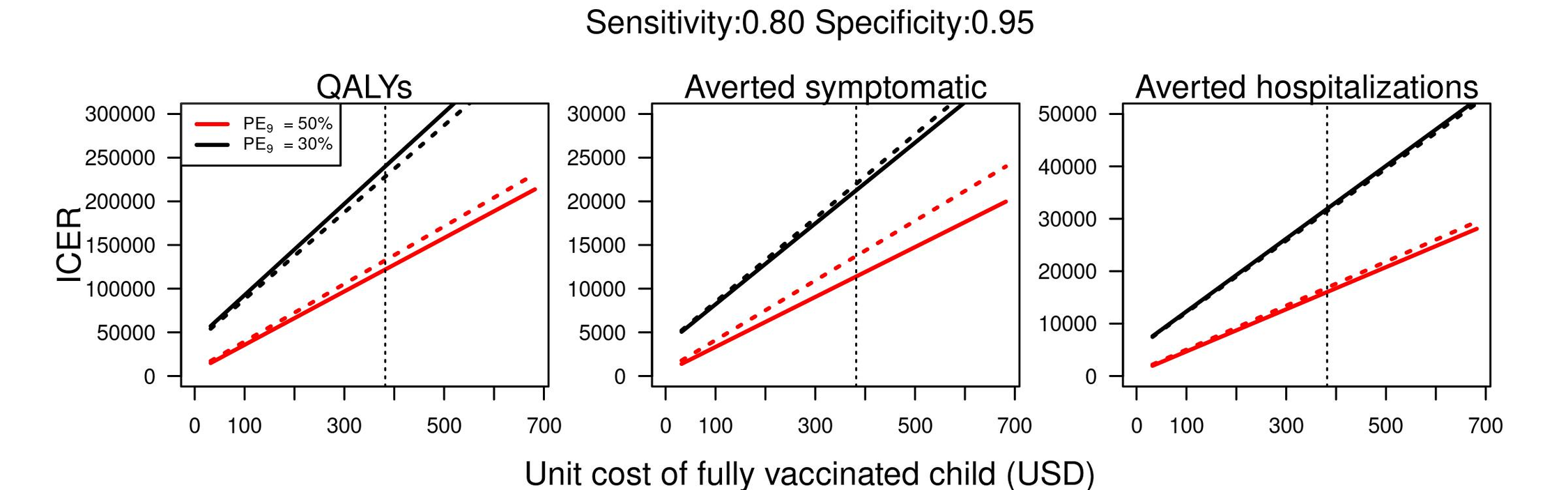

Supplement: S4 Fig — Solid lines shows the baseline scenario of coverage (80%) and dashed line shows lower coverage assumption (50%). All costs in 2019 USD. (TIF) [file pntd.0009606.s005.tif]
