# Supplementary material for: Cost-effectiveness of dengue vaccination in Puerto Rico
Source: PLoS Negl Trop Dis. 2021 Jul 26;15(7):e0009606. doi: 10.1371/journal.pntd.0009606 (PMC8341694; doi:10.1371/journal.pntd.0009606)
Supplement: S1 Table — (DOCX) [file pntd.0009606.s006.docx]

Table S1. Sensitivity to changes in costs and disutility for ICERs measuring costs (2019 USD) per QALYs gained.

| Parameter | $PE_{9}$ | Min | ICER(Min) | Max | ICER(Max) |
| --- | --- | --- | --- | --- | --- |
| costs symptomatic | 50% | 252 | 122,533 | 379 | 121,358 |
| costs hospitalization | 50% | 1,706 | 124,664 | 2,559 | 119,228 |
| unit cost of serological screening | 50% | 1 | 101,068 | 60 | 143,541 |
| disutility symptomatic | 50% | 0.0170 | 139,192 | 0.0917 | 78,538 |
| disutility hospitalization | 50% | 0.0241 | 131,241 | 0.0960 | 87,609 |
| costs symptomatic | 30% | 252 | 240,597 | 379 | 239,433 |
| costs hospitalization | 30% | 1,706 | 242,749 | 2,559 | 237,281 |
| unit cost of serological screening | 30% | 1 | 184,912 | 60 | 297,008 |
| disutility symptomatic | 30% | 0.0170 | 274,684 | 0.0917 | 151,714 |
| disutility hospitalization | 30% | 0.0241 | 257,317 | 0.0960 | 172,741 |
