# Supplementary material for: Cost-effectiveness of dengue vaccination in Puerto Rico
Source: PLoS Negl Trop Dis. 2021 Jul 26;15(7):e0009606. doi: 10.1371/journal.pntd.0009606 (PMC8341694; doi:10.1371/journal.pntd.0009606)
Supplement: S2 Table — (DOCX) [file pntd.0009606.s007.docx]

Table S2. Estimated price for CYD-TDV based on age range, recombinant technology, and recent approval.

|  | Cost/dose | Cost/ 3 doses |
| --- | --- | --- |
| Estimated cost per dose based on three factors (age range, recombinant technology, recent approval): | 107.28 | 321.8 |
| Lowest observed: | 10.85 | 32.6 |
| Highest observed: | 227.93 | 683.8 |
| Average private sector cost per dose of vaccines recommended for 7 to 15 year olds: | 113.54 |  |
| Average private sector cost per dose of recombinant vaccines: | 85.13 |  |
| Average private sector cost of vaccines approved by FDA since 2014: | 123.16 |  |
