# Supplementary material for: Cost-effectiveness of dengue vaccination in Puerto Rico
Source: PLoS Negl Trop Dis. 2021 Jul 26;15(7):e0009606. doi: 10.1371/journal.pntd.0009606 (PMC8341694; doi:10.1371/journal.pntd.0009606)
Supplement: S3 Table — (DOCX) [file pntd.0009606.s008.docx]

Table S3. List of vaccines recommended for 7 to 15 year-olds**^[[1]](#footnote-2)^**.

| Vaccines recommended for 7 to 15 year olds (i.e., close in age to 9 year olds) | Private sector cost/dose |
| --- | --- |
| Meningococcal ACWY-D, ACWY-CRM |  |
| Menveo | 130.75 |
| Menactra | 122.31 |
| Tetanus, diphtheria, & acellular pertussis |  |
| Boostrix | 41.19 |
| Adacel | 45.5 |
| Human papillomavirus |  |
| Gardasil9 | 227.931 |
| **Average** | **113.54** |

1. Source: All Private sector cost/dose from the Vaccine Price List
    (https://www.cdc.gov/vaccines/programs/vfc/awardees/vaccine-management/price-list/index.html) last updated on 12/2/2019 [↑](#footnote-ref-2)
