# Supplementary material for: Cost-effectiveness of dengue vaccination in Puerto Rico
Source: PLoS Negl Trop Dis. 2021 Jul 26;15(7):e0009606. doi: 10.1371/journal.pntd.0009606 (PMC8341694; doi:10.1371/journal.pntd.0009606)
Supplement: S4 Table — (DOCX) [file pntd.0009606.s009.docx]

Table S4. List of vaccines with similar technology as CYD-TDV

| Vaccines that are subunit/recombinant technology (i.e., similar technology as Dengvaxia) | Private sector cost/dose |
| --- | --- |
| Hib (Haemophilus influenzae type b) |  |
| PedvaxHIB | 26.333 |
| ActHIB | 16.51 |
| Hiberix | 10.85 |
| Hepatitis B |  |
| Engerix B | 23.72 |
| Recombivax HB | 23.95 |
| Human papillomavirus |  |
| Gardasil9 | 227.931 |
| Pertussis |  |
| Boostrix | 41.19 |
| Adacel | 45.5 |
| Pneumococcal |  |
| Prevnar 13 | 188.26 |
| Pneumovax | 105.194 |
| Meningococcal |  |
| Menveo | 130.75 |
| Menactra | 122.31 |
| Shingles |  |
| Shingrix | 144.2 |
| **Average** | **85.13** |
