# Supplementary material for: Cost-effectiveness of dengue vaccination in Puerto Rico
Source: PLoS Negl Trop Dis. 2021 Jul 26;15(7):e0009606. doi: 10.1371/journal.pntd.0009606 (PMC8341694; doi:10.1371/journal.pntd.0009606)
Supplement: S5 Table — (DOCX) [file pntd.0009606.s010.docx]

Table S5. List of vaccines introduced in the last 5 years^[[1]](#footnote-2)^.

| Vaccines introduced in the last 5 years (i.e., similar time-frame) | private sector cost/dose | FDA approval |
| --- | --- | --- |
| Shingrix | 144.2 | 2017 |
| Heplisav-B | 115.75 | 2017 |
| Hiberix | 10.85 | 2016 |
| Vaxchora |  | 2016 |
| Bexsero | 170.75 | 2015 |
| Quadracel | 53.13 | 2015 |
| Fluad |  | 2015 |
| Trumenba in the U.S. to prevent serogroup B meningococcal disease | 139.52 | 2014 |
| Gardasil 9 (Merck) | 227.931 | 2014 |
| **Average** | **123.16** |  |

1. Sources: All cost per dose information comes from the VFC vaccine price list (https://www.cdc.gov/vaccines/programs/vfc/awardees/vaccine-management/price-list/index.html). All FDA approval information comes from immunize.org timeline (https://www.immunize.org/timeline/) [↑](#footnote-ref-2)
