# Supplementary material for: Cost-effectiveness of dengue vaccination in Puerto Rico
Source: PLoS Negl Trop Dis. 2021 Jul 26;15(7):e0009606. doi: 10.1371/journal.pntd.0009606 (PMC8341694; doi:10.1371/journal.pntd.0009606)
Supplement: Attachment — Submitted filename: manuscript_dengvaxia_puerto_rico_response_to_reviewers_R2.pdf [file pntd.0009606.s012.pdf]

Response to Reviewers

We thank the editor for the thoughtful and constructive comments. We have addressed each of the comments and have elaborated a response for them. Please see below our point by point response for each of the reviewers' concerns with line numbers referring to the PDF version of the report with changes marked in blue(additions) and red(deletions).

Editor comments

1. *The paper presents no evidence that there is a test licensed for use in Puerto Rico addresses these shortcomings. If there is such evidence, I believe the authors should include it in their manuscript. If such evidence does not exist, then I suggest the authors consider acknowledging the constraint and adjusting their manuscript accordingly. They may wish to consider one or more of the following adjustments: First, they could consider their base values as aspirational, so their base values would apply if an when a "Rapid Vaccination Dengue Test" were licensed and available. Second, they could offer and justify more modest values of sensitivity and specificity if existing tests were repurposed for this pre-vaccination testing despite their limitations. Third, they could consider laboratory-based tests instead of rapid tests. These would be more accurate but entail added costs, logistical challenges, and likely lower vaccination rates.*

   Response: A recent evaluation of commercial dengue virus IGG tests for pre-vaccination screening showed values that are within our range of possibilities evaluated in this study. This study has been presented at the ACIP meeting in February 2021 (`https://www.cdc.gov/vaccines/acip/meetings/downloads/slides-2021-02/24-25/Dengue-Medina.pdf`) [1]. We added this reference to support our chosen values in lines **147- 148**.

   "Similar values were found by the dengue branch surveillance and research laboratory"

2. *19 It would be helpful to express the iatrogenic additional dengue hospitalizations on the same scale (per 1000 population) as the hospitalizations averted.*

   Response: We made the change suggested by the editor and reported the number of additional iatrogenic hospitalizations per 1,000 people in lines **13- 16**.

3. *52 A stronger statement would be that other candidates are showing promising results.*

   Response: We made this change in lines **43- 44**. *"Currently, one dengue vaccine (CYD-TDV) is available and other vaccine candidates are showing promising results"*

4. *60 a vaccine program*

   Response: We made this change (line **52**).

5. *94 in other studies. Note: age 9 was also considered in reference 13*

   Response: We added reference 13 (line **86**).

6. *110 "many times" – four times would be more precise*

   Response: Changed to "four times" (line **102**).

7. *121 consider rewording the sentence to clarify that "compared to those not vaccinated" applies to the entire sentence.*

   Response: We now modified this sentence for clarity (lines **110 - 113**). *"These assumptions are supported by empirical evidence showing that, compared to those not vaccinated, seronegative individuals vaccinated with CYD-TDV have a higher risk of hospitalization after a natural infection, whereas seropositive individuals vaccinated have a lower risk of hospitalization."*

8. *142 To facilitate comparison, it would also be helpful to report the additional hospitalizations per 1,000 population.*

   Response: AS suggested by the reviewer, we now include the number of additional hospitalizations per 1,000 population (lines **139 140** and **244- 247**).

9. *153 See discussion above on sensitivity and specificity and choice of a rapid test.*

   Response: See response to comment 1. We now cite a reference for an evaluation of commercial tests for pre-vaccination screening strategies, which was presented at the ACIP meeting in February 2021.

10. *154 we (lower case) assumed*

    Response: Changed to lower case (line **149**).

11. *329 [Zeng] omitted the cost of screening. Suggest changing to: "simulated universal screening without screening."*

    Response: Thank you for the suggestion. We have incorporated it in the manuscript in lines (**315- 316**).

12. *358 it would be unfeasible (suggest changing to "infeasible.")*

    Response: We made this change (line **344**).

13. *446 Consider updating the citation as this paper is now published.*

    Response: We updated this reference.

14. *468 cost-benefit thresholds*

    Response: We updated this reference.

References

[1] Freddy A. Medina and Jorge Muñoz. *Evaluation of Commercial Dengue Virus IGG Tests for Pre-Vaccination Screening*. Feb. 2021.
