# Supplementary material for: Cost-effectiveness of dengue vaccination in Puerto Rico
Source: PLoS Negl Trop Dis. 2021 Jul 26;15(7):e0009606. doi: 10.1371/journal.pntd.0009606 (PMC8341694; doi:10.1371/journal.pntd.0009606)
Supplement: Attachment — Submitted filename: manuscript_dengvaxia_puerto_rico_response_to_reviewers_R1.docx [file pntd.0009606.s011.docx]

# Response to Reviewers

We thank the reviewers for their thoughtful and constructive comments. We have addressed each of the comments and have elaborated a response for them. Please see below our point by point response for each of the reviewers’ concerns with line numbers referring to the PDF version of the report with changes marked in **blue(additions)** and **red(deletions)**.

# Reviewer 1

1. *The analysis presented matches the plan. The results are presented with good clarity– a few suggestions are included in this review. One aspect of the analysis is omitted from discussion, which is the case in which prevalence of Dengue at or below 9 years of age is greater than 50%. It’s probably wise to limit the options presented in detail for discussion, but would seem useful to included some mention of it.*

- **Response:**
- We have briefly described the impact of higher transmission intensity in Puerto Rico in lines 267 - 269.

2. *The conclusions are supported by the analysis. Limitations of the analysis are described clearly, though one should be called out in the introduction and methods more clearly: the use of government costs only as the ’cost’ component definitely skews the cost-benefit analysis in an unfavorable way.*

- Public health relevance is addressed, particularly the very important point that the specificity of tests applied prior to vaccination greatly alters the risks and benefits of the vaccination strategy.
- **Response:**
- We have now explained our choice of economic perspective in lines 154 - 157, as well as its potential underestimation of the cost-effectiveness under other perspectives.

3. *The authors state that the code will be available on GITHUB. This is excellent, but from the statement itself, it’s not clear that this satisfies the requirement that the data be made available. It appears that all data are from public sources and most or all are given within the manuscript as well, in which case this could simply be stated.*

- **Response:**
- Data sources to parameterize the model are publicly available with references to the sources described in the methods.

4. *Line 47, spell-check screening*

- **Response:**
- We fixed this typo (line 53).

5. *Line 102, it would seem that evidence for the other side of this coin should also be cited here to complete the argument—that is, the effect of vaccination after prior infection. This seems to be in Table 1.*

- **Response:**
- We have included that side of the argument in that sentence now (lines 111 - 112).

6. *Line 236, “In the baseline scenario of costs”…. Please spell this baseline out again, so the reader doesn’t have to go back through the manuscript to find it and differentiate from what follows.*

- **Response:**
- We modified this sentence as suggested by the reviewer (lines 245 - 248).

7. *Line 244 – In terms OF?*

- **Response:**
- Fixed typo.

8. *Line 315 – The point about varying effects depending upon the dominance, year to year, of different strains, is a good one. It would be enlightening to discuss this a bit further—is there a worst-case scenario and best-case scenario that could be described, at least in general terms? It would seem that the worst-case scenario is worth discussing.*

- **Response:**
- We have added a discussion about this subject in lines 338 - 342. Even though it is unfeasible to make projections about future dominance of serotypes, our model projections would be robust if vaccine efficacy were homogeneous across serotypes. With heterogeneous vaccine efficacy across serotypes, our results could overestimate or underestimate the benefits of vaccination, depending on the dominant serotype.

9. *General – PE9 = 60% was simulated but is not discussed at all, presumably for simplicity. A statement that ICER would be even more favorable at 60% and a pointer to the figures/tables that are relevant would seem to be appropriate.*

- **Response:**
- For simplicity, we focused on the 30% and 50% as our main analysis and explored the alternative analysis of 60% in the supplementary material. We have now added a short description of the results in the main text in lines 241 - 243. In addition, we briefly presented these results in the discussion in lines 267 - 269.

10. *General – The limitation of the costs to what the government pays, rather than total societal costs, while reasonable, should be very clearly stated in the introduction and methods, as it significantly decreases the figures used for economic benefit and skews the results of the paper toward an unfavorable decision about vaccination.*

- **Response:**
- As mentioned in our response to comment 9 above, we have now explained our choice of economic perspective in lines 154 - 157, as well as its potential underestimation of the cost-effectiveness under other perspectives.

11. *Ref 10. Is the author correct?*

- **Response:**
- We have fixed this typo.

# Reviewer 2

1. *Line 74: Please describe what is he status quo when discussing agenting-based model. It is not clear whether the status quo was the scenario without vaccine or the scenario with vaccination but without screening.*

- **Response:**
- The status quo scenario in our analysis is the scenario without vaccine. We have specified that in line 124.

2. *Line 90: Mean prevalence was set at 50%. However, lower and upper bound was set as 0.3 and 0.6, respectively. Please clarify why authors assume a potential asymmetrical distribution of the prevalence.*

- **Response:**
- Our baseline assumption of prevalence was 50%. Because of the potential negative impacts of the vaccine in lower transmission settings, we chose to simulate a scenario of lower prevalence (30%). As an alternative scenario, we simulated a slightly higher prevalence of 60%, which we describe in the supplementary material (lines 94 - 100). The upper value of 60% was informed by a recent unpublished study provided by the CDC branch in which they found that 60% seroprevalence in 9 year olds was the maximum estimate in Puerto Rico.

3. *Line 143 mentioned that the study was conducted from a public health perspective. If I understood correctly, the authors meant to say “health system’s perspective” or “health providers perspective”. According to recommendations for conduct cost-effectiveness analyses in health (*[*https://jamanetwork.com/journals/jama/article-abstract/2552214*](https://jamanetwork.com/journals/jama/article-abstract/2552214)*), it is often recommended to conduct CEA from both societal and health system’s perspectives. Authors needs to further explain this perspective on what has been excluded or included in this perspective. It is not until line 177 did I realize the premature death was accounted for in this study.*

- **Response:**
- Based on our understanding, in our study, “public health perspective” and “health care sector perspective” would be considered the same. Both perspectives include medical costs, vaccination costs, and health outcomes of the population of interest. While there are recommendations to use both the health care sector and societal perspectives, other schools of thought argue that either/both/neither of those perspectives should be requirements for a CEA ( [https://www.ncbi.nlm.nih.gov/pmc/articles/PMC6038124/](./%20https://www.ncbi.nlm.nih.gov/pmc/articles/PMC6038124/)). For example, some CEA studies use the patient perspective because the patient is the most relevant decision maker for some decisions, these studies might not calculate a healthcare sector or societal perspective estimate.
- To address the reviewer’s comment, we have now explained in lines 154 - 157 that we chose this perspective because we are hoping to inform decision makers in the public health sector. For those decision makers, the healthcare sector or public health perspective would be appropriate. We have also listed the costs included in the analysis in lines 152 - 154.

4. *Line 153: The cost of vaccine varies substantially; it could be as low as 32.6 to as high as 683.8. The cost of vaccine would be a major determinations of the cost-effectiveness. It would be helpful to explain why there was such a huge variation in Puerto Rico in the discussion section. Will the group purchasing would affects the procurement price of vaccine?*

- **Response:**
- See response in the next comment.

5. *it is not clear how $382 was derived?*

- **Response:**
- At this point in time, the cost of the vaccine is unknown. Consequently, we expressed this uncertainty by including a wide range of the potential cost of the vaccine based on other vaccines in the United States. We estimated the cost per dose based on three factors: age range, recombinant technology, and recent approval. The average price of these private sector vaccines per dose was 107 USD. Hence, we set the baseline value to 382 (3 doses). For the range of costs for the vaccine that we considered, the lowest value for the vaccines listed in Tables S2-S4, was 10 USD (30 USD for three doses). The highest value from the vaccines included in the analysis was 227 USD for one dose, so we set the maximum value to 682 for three doses. We have briefly described the process to calculate the cost of the vaccine in lines 163 - 168.

6. *Study shows that dengue seroprevalence varies among municipalities (*[*https://journals.plos.org/plosntds/article?id=10.1371/journal.pntd.0002159*](https://journals.plos.org/plosntds/article?id=10.1371/journal.pntd.0002159)*). Given that the prevalence and sensitivity and specific of screen would affect the cost-effectiveness of the vaccine, it would be helpful to discuss the implication of the strategy of implementation DENV in Puerto Rico in municipalities with different seroprevalence.*

- **Response:**
- The study cited by Sharp et al shows that the overall and dengue serotype specific incidence of clinical reported illness from passive and enhanced surveillance in 2 municipalities during the 2010 Puerto Rico dengue epidemic varied by municipality, and did not include data on seroprevalence by municipality. Comprehensive seroprevalence data by municipality is not available in Puerto Rico. Available data suggests that dengue seroprevalence in 9-16 year olds in urban areas of Puerto Rico is roughly 50%. Dengue is reported from every municipality during outbreaks (note Figure 2 in the reviewer cited paper), but incidence and presumably seroprevalence is lower in some mountainous and relatively thinly populated municipalities in the center of the island. The vast majority of children in Puerto Rico live in urban areas and an implementation strategy which prioritizes such areas with more children and likely higher dengue seroprevalence would be a rational approach. Ideally, dengue seroprevalence should be measured in less densely populated municipalities to assess safety and cost effectiveness more precisely for these areas. However, even if age specific seroprevalence in children in less populated rural areas is half that of children in urban areas, use of a highly specific screening test (e.g. 98%) would still result in high predictive value of a positive test (>90%), and a relatively cost effective program based on the model presented.
- We have now added a discussion about this subject in lines 326 - 334, which we include below.
- “In addition, we assumed that prevalence was similar across regions of Puerto Rico based on studies of seroprevalence. Although comprehensive seroprevalence data by municipalities in Puerto Rico is not currently available, less populated municipalities would likely have lower prevalence than densely populated urban areas. In consequence, our assumption of $PE_{9}=50\text{\%}$ would likely cover the majority of children who live in densely populated areas. Ideally, dengue seroprevalence studies could be performed to measure prevalence in less densely populated areas before introduction of the vaccine. Nonetheless, our results show that this intervention has epidemiological and economic benefits even in lower transmission scenarios ($PE_{9}=30\text{\%}$), as long as specificity of screening is high.”
